# SRSF9 promotes cell proliferation and migration of glioblastoma through enhancing CDK1 expression

**DOI:** 10.1007/s00432-024-05797-0

**Published:** 2024-06-06

**Authors:** Chunyuan Luo, Juan He, Yang Yang, Ke Wu, Xin Fu, Jian Cheng, Yue Ming, Wenrong Liu, Yong Peng

**Affiliations:** 1 Laboratory of Molecular Oncology, Frontiers Science Center for Disease-Related Molecular Network, State Key Laboratory of Biotherapy, West China Hospital, Sichuan University, Chengdu, 610041 China; 2 Department of Neurosurgery, West China Hospital, Sichuan University, Chengdu, 610041 China

**Keywords:** SRSF9, Glioblastoma, CDK1, Proliferation, Migration

## Abstract

**Background:**

Glioblastoma (GBM) is a highly aggressive and prevalent brain tumor that poses significant challenges in treatment. SRSF9, an RNA-binding protein, is essential for cellular processes and implicated in cancer progression. Yet, its function and mechanism in GBM need clarification.

**Methods:**

Bioinformatics analysis was performed to explore differential expression of SRSF9 in GBM and its prognostic relevance to glioma patients. SRSF9 and CDK1 expression in GBM cell lines and patients’ tissues were quantified by RT-qPCR, Western blot or immunofluorescence assay. The role of SRSF9 in GBM cell proliferation and migration was assessed by MTT, Transwell and colony formation assays. Additionally, transcriptional regulation of CDK1 by SRSF9 was investigated using ChIP-PCR and dual-luciferase assays.

**Results:**

The elevated SRSF9 expression correlates to GBM stages and poor survival of glioma patients. Through gain-of-function and loss-of-function strategies, SRSF9 was demonstrated to promote proliferation and migration of GBM cells. Bioinformatics analysis showed that SRSF9 has an impact on cell growth pathways including cell cycle checkpoints and E2F targets. Mechanistically, SRSF9 appears to bind to the promoter of CDK1 gene and increase its transcription level, thus promoting GBM cell proliferation.

**Conclusions:**

These findings uncover the cellular function of SRSF9 in GBM and highlight its therapeutic potential for GBM.

**Supplementary Information:**

The online version contains supplementary material available at 10.1007/s00432-024-05797-0.

## Introduction

Glioblastoma (GBM) is a highly invasive and malignant brain tumor, representing approximately 49% of all gliomas (Janjua et al. 2021; King and Benhabbour 2021; Schaff and Mellinghoff 2023). Despite aggressive treatment strategies including surgery, radiotherapy, chemotherapy and targeted therapy, the clinical efficacy remains unsatisfactory, with a poor overall survival (Tan et al. 2020; Liu et al. 2023a). Therefore, there is an urgent need to elucidate molecular mechanisms underlying GBM progression and identify precise molecular markers for early prognosis and disease monitoring.

RNA splicing is a fundamental process to regulate gene expression and maintain cellular homeostasis. Serine/arginine-rich splicing factor 9 (SRSF9) is an important component of the spliceosome machinery participating in mRNA splicing (Barta et al. 2010). Increasing evidence demonstrates that SRSF9 regulates progression of different cancers. For example, Fu et al. showed that SRSF9 promotes Wnt signaling-mediated tumorigenesis by increasing β-catenin biosynthesis (Fu et al. 2013b); SRSF9 was reported to participate in colorectal cancer (CRC) progression through stabilizing DSN1 mRNA in an m6A-related manner and to regulate GPX4 expression (Wang et al. 2021, 2022b); Wang et al. (2021) highlighted the role of SRSF9 as a critical driver of colorectal cancer progression and resistance to Erastin-induced ferroptosis. Liu et al. (2022a) reported that SRSF9 is upregulated in GBM and may be a biomarker for GBM, but biological function and molecular mechanism of SRSF9 in GBM remains unclear.

This study investigated the role of SRSF9 in GBM progression and dissected its underlying molecular mechanism. Firstly, we observed the upregulation of SRSF9 expression in GBM tissues comparing to their adjacent normal controls and found that elevated SRSF9 expression correlates with glioma progression and poor patient prognosis. Subsequently, we demonstrated that SRSF9 significantly enhanced the proliferation and migration of glioma cells through both gain-of-function and loss-of-function strategies. Mechanistically, SRSF9 is thought to interact with the promoter of CDK1 gene, leading to elevated transcript levels and increased GBM cell growth. This evidence positions SRSF9 as a potential biomarker and therapeutic target for GBM.

## Materials and methods

### Cell lines and tissues

Human glioblastoma cell lines (U251, LN18, LN229, T98G, A172, U87) and HEK293T cells were cultured in Dulbecco’s Modified Eagle Medium (DMEM) with 10% fetal bovine serum and 1% penicillin/streptomycin, then maintained at 37 °C in a 5% CO_2_ atmosphere. Glioblastoma tumors and their adjacent normal tissues were collected from West China Hospital, Sichuan University, with ethical approval and informed patient consent.

### Plasmid construction

To construct the SRSF9 overexpressing plasmid, cDNA was reversely transcribed from total RNA of GBM cells and the open reading frame (ORF) encoding SRSF9 was amplified by Phanta Super-fidelity DNA polymerase (Vazyme, P505-d1), then the ORF fragment was recombinantly inserted into the linear vector pCDH-CMV-MCS-EF1 digested by *EcoR*I and *BamH*I. To knock down SRSF9 or CDK1 expression, the synthesized oligonucleotides were annealed and inserted into the pLKO.1-TRC vector at *Age*I and *EcoR*I sites. To assess CDK1 promoter activity, a fragment of the CDK1 promoter region (chr10:60,778,042–60,780,047; from − 457 to + 1570, relative to the transcription start site of CDK1 gene) was amplified from U251 genomic DNA and inserted into the pGL3-basic vector at *Kpn*I and *Hind*III sites. Primer sequences are provided in Table S1.

### Cell transfection and lentiviral infection

HEK293T cells were transfected with the target plasmid and packaging plasmids psPAX2 and pMD2G in the ratio of 4:3:1, and the supernatants were collected as lentivirus solution at 48 and 72 h. U251, U87, and LN229 cells were infected with the lentivirus in the presence of 8 μg/mL polybrene (Solarbio, H8761). Stable cell lines were established following selection with 2 μg/mL puromycin (Biofroxx, 1299MG025).

### Quantitative real-time PCR (RT-qPCR) and semi-quantitative RT-PCR

Total RNAs from cells and tissues were extracted by RNAiso Plus reagent (TaKaRa, 9108) and reverse-transcribed into cDNAs using the PrimeScript RT Reagent Kit (TaKaRa, RR047A). RT-qPCR was conducted using specific primers on the Applied Biosystems Real-Time PCR System. Semi-quantitative RT-PCR was performed with a thermal cycler (Applied Biosystems, USA), and PCR products were analyzed by agarose gel electrophoresis. Primer sequences are listed in Table S1.

### Western blot

Total proteins were isolated from cells using RIPA lysis buffer (Beyotime Biotechnology, P0013B) containing protease and phosphatase inhibitors (Bimake, B15001) and measured by BCA Protein Assay Kit (Beyotime Biotechnology, P0010). Proteins were separated by sodium dodecyl sulfate–polyacrylamide gel electrophoresis (SDS-PAGE) and transferred to polyvinylidene difluoride membrane (Millipore). The membrane was blocked with 5% skim milk (Biofrox, 1172GR500) for 1 h at room temperature, incubated with primary antibody overnight at 4 °C, washed and incubated with HRP-conjugated secondary antibody for 1 h at room temperature. Protein detection was performed by chemiluminescence on a ChemiDoc imaging system (Bio-Rad). Antibodies: GAPDH (CST, cat#3683, 1:2000); SRSF9 (Proteintech, cat#17926-1-AP, 1:1000); CDK1 (Proteintech, cat#19532-1-AP, 1:1000).

### Cell migration assay

Cells were suspended in 200 µL DMEM with 1% BSA and seeded in the upper chamber of a Transwell insert (Millipore, PI8P01250), and 600 µL DMEM with 5% FBS was added into the lower chamber. After migrating for 16 h at 37 °C in 5% CO_2_ atmosphere, cells that migrated to the bottom of the insert were fixed in 4% paraformaldehyde (Biosharp, BL539A) for 20 min and then stained with 0.5% crystal violet (Beyotime Biotechnology, C0121) for 30 min. Stained cells were photographed and counted under a light microscope.

### Methylthialazole tetrazolium (MTT) assay

To assess cell viability, 800 cells were seeded into 96-well plate and cultured overnight at 37℃. At indicated time points (1, 3, 5, and 7 days), 10 µL of MTT solution was added to each well and incubated at 37 °C for 4 h. After removing supernatants, 150 µL of DMSO was used to dissolve the formazan crystals, and the absorbance was measured at 570 nm using an enzyme marker (BioTek).

### Colony formation assay

For colony formation assay, cells were inoculated into 6-well plates and incubated at 37 °C in 5% CO_2_ for 10 days, changing medium every three days. After incubation, colonies were first washed with PBS, then fixed with 4% paraformaldehyde for 20 min at room temperature and stained with 0.5% crystal violet for 30 min. The stained cells were imaged using a scanner and the dye was dissolved in ethanol and analyzed at 570 nm for quantification.

### Cell cycle analysis

Cells were harvested by EDTA-free trypsin, washed three times with PBS, and then fixed by 70% ethanol at 4 °C overnight. After washing and centrifugation, cells were stained with 50 μg/mL propidium iodide and 100 μg/mL RNase A in PBS. Cell cycle analysis was performed by CytoFLEX flow cytometer and analyzed with ModFit software.

### Measurement of mRNA stability

Cells were treated with 2 μg/mL actinomycin D (Selleck, S8964) for indicated times and then total RNAs were extracted. The stability of CDK1 mRNA was evaluated using RT-qPCR to quantify its half-life.

### Immunofluorescence assay

Tumor tissues were fixed in 4% paraformaldehyde, dehydrated, paraffin-embedded, and then cut into 6 μm-thick sections. Sections were baked at 65 °C for 4 h, dewaxed in xylene, and rehydrated through a graded series of alcohol solutions. Antigen retrieval was performed by microwaving for 8 min in antigen repair solution (Zsbio, ZLI-9065), then cooling, treating with H_2_O_2_, and permeabilizing with Triton X-100. Sections were blocked with 5% goat serum (Zsbio, ZLI-9056) and then incubated with primary antibodies against SRSF9 (Proteintech, cat#17926-1-AP, 1:100) and CDK1 (Proteintech, cat#19532-1-AP, 1:50) overnight at 4 °C, followed by treatment with the secondary antibodies for 1 h at room temperature. Cells were counterstained with DAPI solution and visualized under a fluorescence microscope.

### Chromatin immunoprecipitation (ChIP)

ChIP assays were performed using the SimpleChIP^®^ Plus Enzymatic Chromatin IP Kit (CST, 9004), in which 5 × 10^6 cells per reaction were fixed with 1% formaldehyde to maintain protein-DNA interactions. Chromatin was digested into 150–900 bp by Micrococcal Nuclease digestion. Immunoprecipitation was performed using ANTI-FLAG^®^ M2 affinity gel (Sigma, cat#A2220) to enrich Flag-tagged SRSF9 complexes. To assess the efficiency of immunoprecipitation, one-fifth of the samples were analyzed by Western blot with anti-Flag antibody (CST, cat#14793). The remaining four-fifths of the samples were subjected to reversal of cross-links, DNA purification and semi-quantitative PCR to identify the association of SRSF9 protein with  the CDK1 promoter .

### Dual-luciferase reporter assay

HEK293T cells were transfected with the CDK1 luciferase reporter plasmid (pGL3-basic-CDK1), pRL-TK Renilla, and SRSF9-expressing plasmid via Lipofectamine 2000 (Invitrogen). After 36 to 48 h, luciferase activities were measured using a Dual-luciferase Reporter Assay Kit (Promega, E1910) with a microplate reader (PerkinElmer), and the Firefly/Renilla ratio was calculated to assess the effect of SRSF9 on CDK1 promoter activity.

### Bioinformatics analysis methods

#### Gene expression analysis

Data from the TCGA database (https://www.cancer.gov/ccg/research/genome-sequencing/tcga) were utilized to analyze and visualize SRSF9 expression in various cancers. We examined SRSF9 and CDK1 mRNA levels in GBM and adjacent normal samples using databases such as BEST (https://rookieutopia.com/) and GEPIA2 (Tang et al. 2019) (http://gepia2.cancer-pku.cn/), and assessed protein levels through the UALCAN database (http://ualcan.path.uab.edu/). SRSF9 expression in different grades of GBM was analyzed using the CGGA database (http://www.cgga.org.cn/). In addition, we analyzed the correlation between SRSF9 and CDK1 expressions in GBM using our own RNA-seq data from 38 paired tumor and adjacent normal tissues, collected at West China Hospital, Sichuan University.

#### Pathway enrichment analysis

Biomarker Exploration of Solid Tumors (BEST) provides access to 2,277 GBM samples. Through BEST, we conducted a GSEA enrichment analysis on SRSF9, utilizing two datasets: one based on KEGG, termed GSEA-KEGG analysis, and another based on the Hallmark dataset, named GSEA-Hallmark analysis. Furthermore, we analyzed the correlation of SRSF9 expression with cell cycle, E2F targets, and G2M checkpoint.

#### Survival curve analysis

The prognostic relevance of SRSF9 expression in various cancers was assessed using the Kaplan–Meier Plotter database (http://kmplot.com/analysis/), which includes data from GEO, EGA, and TCGA. The correlation between SRSF9 mRNA levels and overall survival (OS) in the GBM group was calculated by the Kaplan–Meier curve and log-rank test, a tool that automatically generates risk ratios (HR) and 95% confidence intervals, with a *p *value of < 0.05 considered statistically significant. In addition, we investigated the prognostic significance of SRSF9 or CDK1 expression within BEST to explore their potential impact on survival outcomes in GBM patients.

### Statistical analysis

Statistical analyses in our study were conducted using GraphPad Prism 8.0. Quantitative data were evaluated using two-tailed Student’s *t*-test, one-way or two-way ANOVA. Statistical significance was determined at *p < 0.05, **p < 0.01, ***p < 0.001, with "ns" indicating non-significant results.

## Results

### Expression and prognostic significance of SRSF9 in glioblastoma and other cancers

**Fig. 1:**
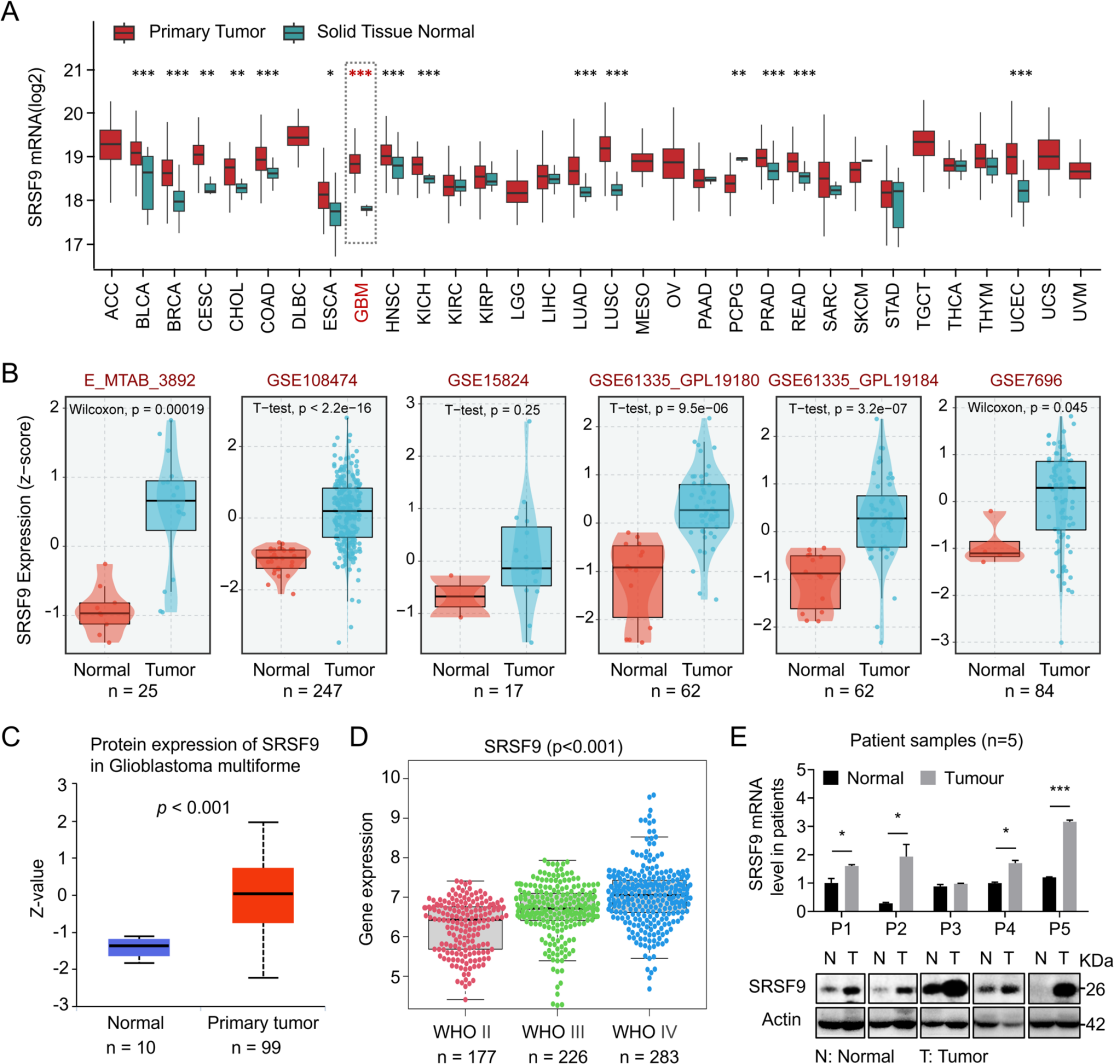
SRSF9 expression is abnormally high in GBM. **A** SRSF9 expression across different tumor types. **B** SRSF9 expression analyzed in six independent GEO cohorts. **C** Box plot of SRSF9 protein expression in GBM (99 samples) compared to normal tissues (10 samples) in UALCAN data (Li et al. 2023). **D** Analysis of SRSF9 expression across various grades of GBM using Chinese Glioma Genome Atlas (CGGA) database. **E** Western Blot and RT-qPCR assessments of SRSF9 protein and mRNA expression in GBM patient tissues

A comprehensive analysis of SRSF9 mRNA levels in TCGA database identified significant upregulation of SRSF9 in various cancers, notably in GBM, breast cancer, and lung squamous cell carcinoma, with tumor tissues exhibiting markedly higher levels than adjacent normal tissues (Fig. 1A, B). The elevated SRSF9 expression in GBM tumor tissues was also validated at protein level using UALCAN datasets (Fig. 1C), and a stage-specific increase in SRSF9 expression correlated to advancing GBM stages according to WHO grading (Fig. 1D). RT-qPCR and Western blot analyses further confirmed the elevated expression of SRSF9 in GBM samples compared to adjacent normal tissues collected by us (Fig. 1E). These findings indicate a strong association between increased SRSF9 expression and glioma progression.

**Fig. 2:**
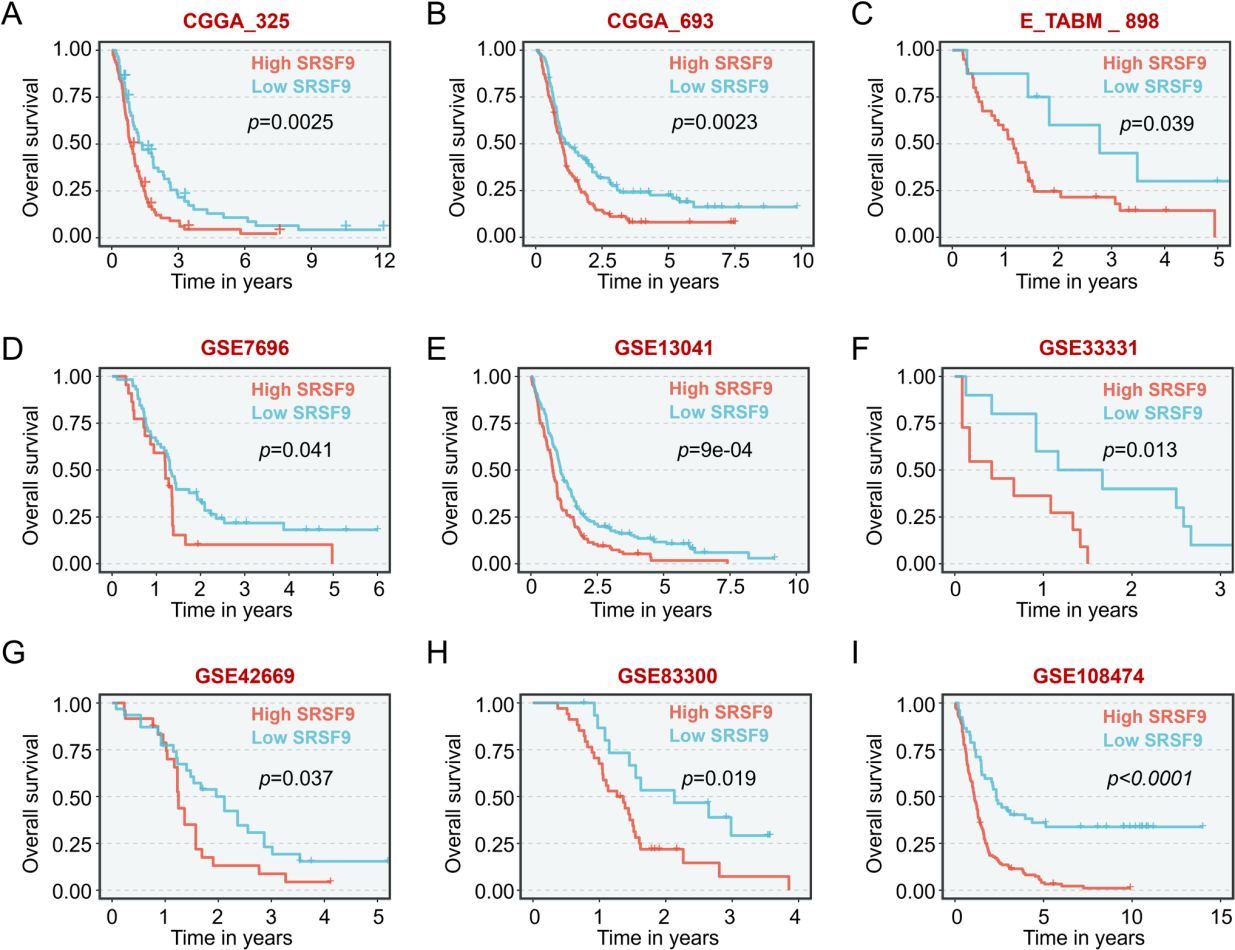
Higher SRSF9 expression exhibited significantly poorer overall survival in GBM across various datasets: **A** CGGA_325 (*n* = 137), **B** CGGA_693 (*n* = 237), **C** E_TABM_898 (*n* = 48), **D** GSE7696 (*n* = 80), **E** GSE13041 (*n* = 267), **F** GSE33331 (*n* = 21), **G** GSE42669 (*n* = 55), **H** GSE83300 (*n* = 50), and **I** GSE108474 (*n* = 195)

To evaluate SRSF9’s prognostic potential, we employed the data from CGGA (Zhao et al. 2021) and E_TAMB_898 cohorts to perform Kaplan–Meier analyses. The results showed that higher SRSF9 expression was consistently correlated to shortened overall survival of glioma patients (Fig. 2A–C), which was further validated by 6 GEO cohorts for GBM patients (Fig. 2D–I). In addition, such correlation was also observed in other cancers, such as lung adenocarcinoma and hepatocellular carcinoma (Fig. S1).

### SRSF9 promotes proliferation and migration of GBM cells

**Fig. 3:**
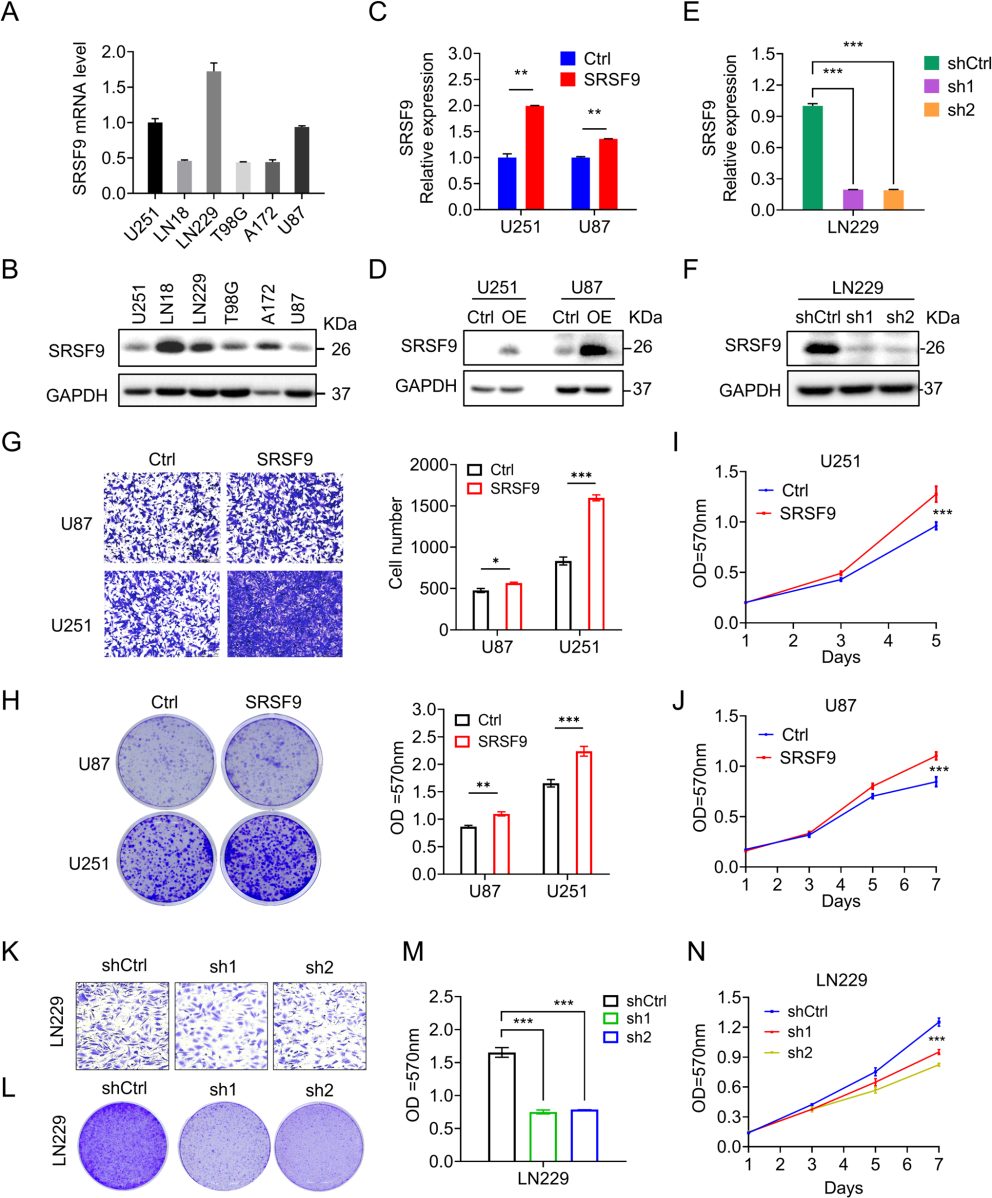
SRSF9 promotes proliferation and migration of GBM cells. **A** SRSF9 mRNA levels in GBM cells were measured using RT-qPCR. **B** Western blot assay was used to detect SRSF9 protein levels in six GBM cell types. **C**, **D** Overexpression of SRSF9 in U251 and U87 stable cell lines was confirmed by RT-qPCR and Western blot analyses. **E**, **F** Knockdown of SRSF9 expression in LN229 was verified by RT-qPCR and Western blot analyses. **G**, **K** Transwell assays to show the effect of SRSF9 on cell migration. **H**, **L**, **M** Colony formation assays to assess the effect of SRSF9 on cell growth. **I**, **J**, **N** MTT assays to demonstrate the effect of SRSF9 on cell proliferation

To explore the cellular function of SRSF9 in GBM, we measured SRSF9 expression among different GBM cell lines (Fig. 3A, B), and then examined its effects on cell proliferation and migration.

The gain-of-function experiments were conducted in U251 and U87 cells with relatively low SRSF9 expression, and the stable overexpression of SRSF9 in these cells were obtained after successfully infecting cells with SRSF9 overexpression viruses and confirmed at both mRNA and protein levels (Fig. 3C, D). Subsequently, Transwell assays showed enhancement of GBM cell migration by SRSF9 (Fig. 3G), while MTT and colony formation assays indicated its role in promoting cell proliferation (Fig. 3H–J).

In addition to overexpression, knockdown experiments were conducted to confirm the SRSF9 function in GBM, for which two shRNAs targeting SRSF9, named sh1 and sh2, and a control shRNA (shCtrl) were designed to prepare lentiviruses. After infecting the high SRSF9-expressing cell line LN229 with these shRNA viruses, a notable reduction in SRSF9 expression was observed at both mRNA and protein levels (Fig. 3E, F). The knockdown of SRSF9 expression led to a decreased cell migration (Fig. 3K) and a reduced cell proliferation (Fig. 3L–N). Taken together, SRSF9 promotes proliferation and migration of GBM cells.

### Impact of SRSF9 on cell cycle and DNA replication pathways in GBM

**Fig. 4:**
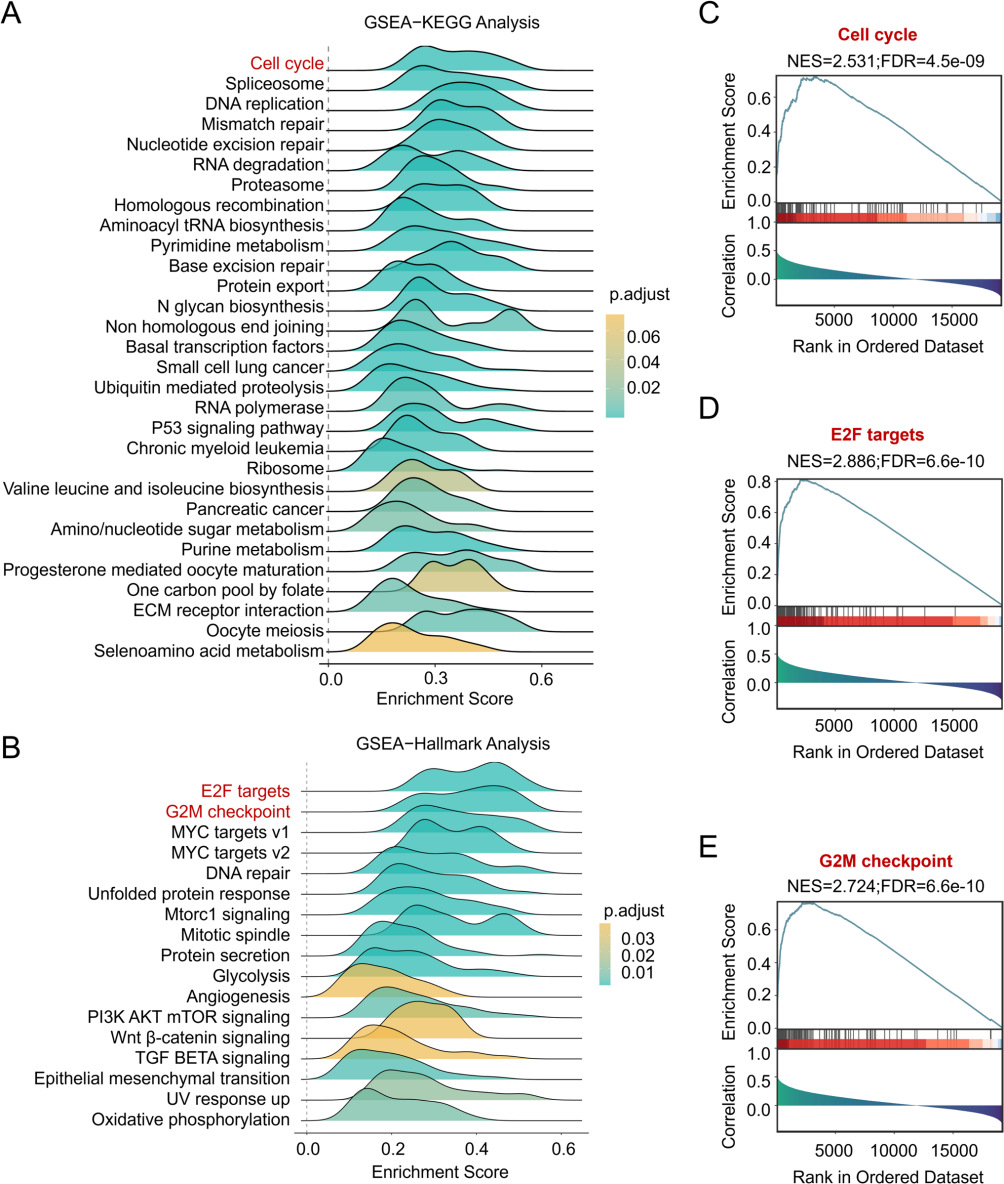
Potential SRSF9-driven signaling pathway in GBM. **A**, **B** SRSF9 signaling pathway engagement in GBM as shown by GSEA-KEGG and GSEA-Hallmark. The *x*-axis represents the enrich score of pathways in the data, while color gradients denote statistical significance through adjusted p-values. **C**–**E** The relationship between SRSF9 expression and multiple pathways, as indicated by GSEA enrichment scores, highlighting its significance in GBM pathogenesis

To elucidate the mechanism by which SRSF9 promotes GBM progression, we performed bioinformatics analyses to predict potential pathways affected by SRSF9 using the data from Biomarker Exploration of Solid Tumors (BEST) (Liu et al. 2023b). Gene Set Enrichment Analysis(GSEA)—Kyoto Encyclopedia of Genes and Genomes (KEGG) analysis suggested that SRSF9 is related to critical pathways in cell cycle, spliceosome, and DNA replication (Fig. 4A). Furthermore, GSEA-Hallmark analysis showed SRSF9’s association with E2F targets, G2/M checkpoint, and mitotic spindle elements (Fig. 4B). Notably, the pathways governing cell growth, including cell cycle, G2M checkpoints and E2F targets, were particularly abundant in GBM with elevated SRSF9 expression, indicating its strong influence on proliferation and tumorigenesis (Fig. 4C–E).

### Positive correlation of SRSF9 upregulation with CDK1 in GBM

**Fig. 5:**
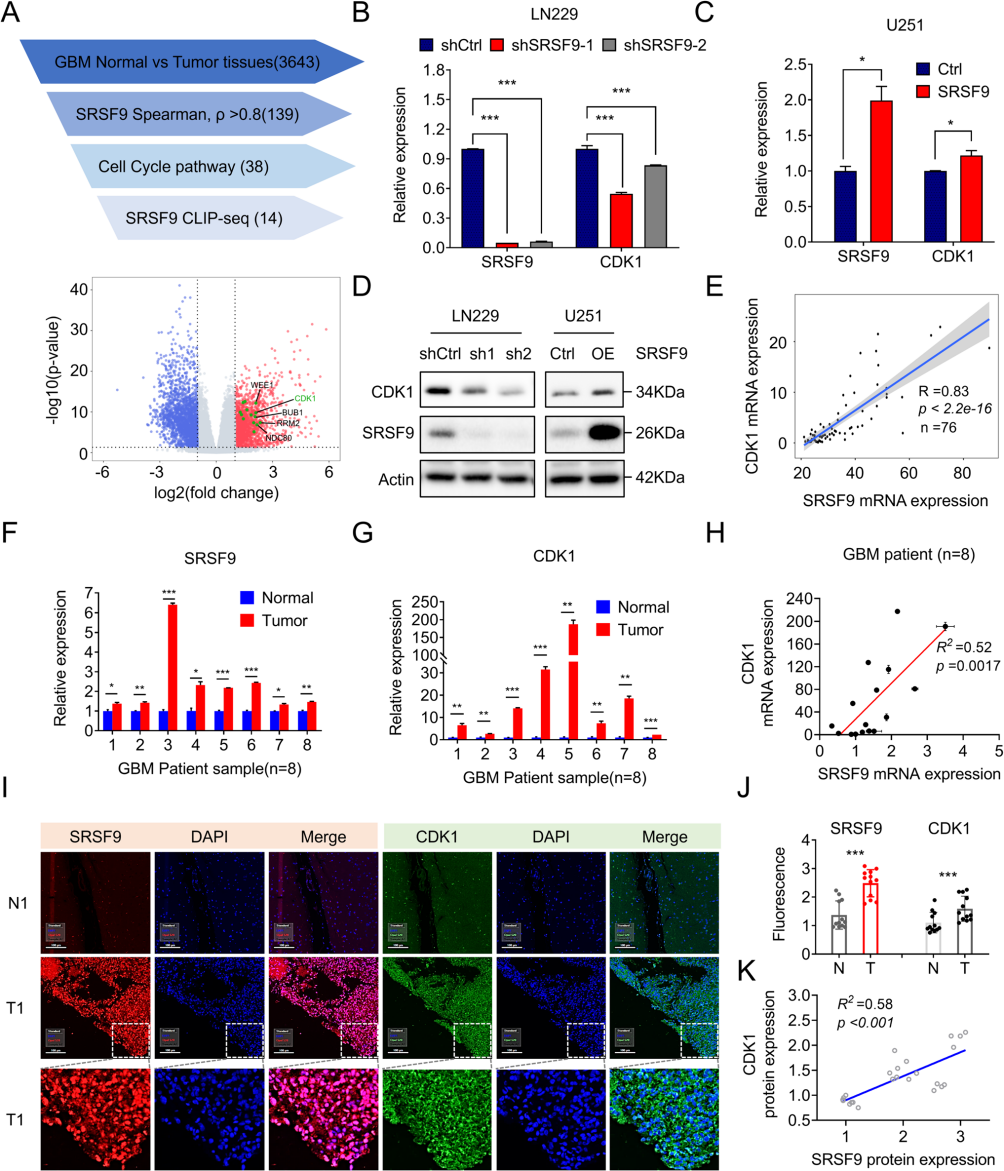
SRSF9 upregulation and positive correlation with CDK1 in GBM. **A** Identification of functionally important targets within the SRSF9 axis through screening strategies (top), and the illustration of their differential expression in GBM tumors versus peritumor tissues using volcano plots (bottom). **B**, **C** RT-qPCR assay quantified CDK1 mRNA levels in GBM cells with altered SRSF9 expression. **D** Western blot analysis revealed CDK1 protein levels after SRSF9 overexpression or knockdown. **E** A positive correlation between SRSF9 and CDK1 expression in GBM was analyzed using data described in the method Sect. 2.14.1 (*n* = 76). **F**, **G** RT-qPCR analyzed SRSF9 and CDK1 in 8 paired GBM and adjacent normal tissues. **H** Correlation analysis confirmed a significant association between SRSF9 and CDK1 in GBM tissues (*n* = 8). **I**–**K** Immunofluorescence assays quantified the levels of SRSF9 and CDK1 in normal and GBM tissues, capturing fluorescence intensities in images for correlation analysis (*n* = 3)

To further understand the molecular mechanism by which SRSF9 exerts its tumor-promoting role in GBM, we employed various strategies and screened out key SRSF9-regulated genes that influence cell cycle. Our screening strategy commenced with the analysis of 3643 differentially expressed genes between GBM and adjacent tissues (Table S2, |log_2_ FC|> 1, *p *value < 0.05), refined through Spearman correlation to identify 139 strongly SRSF9-correlated genes (Table S3, *ρ* > 0.8). Further focusing on cell cycle gene set (Subramanian et al. 2005; Liberzon et al. 2011, 2015) (from MSigDB, Table S4) and CLIP-seq data (Li et al. 2014) (from StarBase, Table S5), we narrowed our findings to 14 key genes potentially interacting with SRSF9, notably including CDK1, as illustrated in our differential expression volcano plots (Fig. 5A). Cyclin-Dependent Kinase 1 (CDK1) is a key regulator of cell cycle progression and its elevated expression participates in tumor development (Ding et al. 2020; Ghafouri-Fard et al. 2022). We found that CDK1 expression is upregulated in GBM at both mRNA and protein levels (Fig. S2), so we wonder if SRSF9 regulates CDK1 expression. To address this issue, we ectopically expressed or knocked down SRSF9 expression. The results showed that overexpression and knockdown of SRSF9 were accompanied by increase and decrease in CDK1 expression, respectively (Fig. 5B–D). Moreover, the significant positive correlation between SRSF9 and CDK1 expression in GBM tissues was observed via analyzing RNA sequencing data (*n* = 76) from our study (Wang et al. 2022a) (*R* = 0.83, *p* < 0.002; Fig. 5E), which was further validated in eight collected GBM clinical samples at mRNA levels (Fig. 5F–H) and protein levels (Fig. 5I–K, Fig. S3). Besides, immunofluorescence staining data also revealed the spatial localization of these proteins: CDK1 was primarily located in the cytoplasm, whereas SRSF9 was mainly found in the nucleus (Fig. 5I–K), suggesting that SRSF9 may regulate CDK1 expression in the nucleus.

### SRSF9 promotes proliferation and migration of GBM cells through enhancing CDK1 expression

**Fig. 6:**
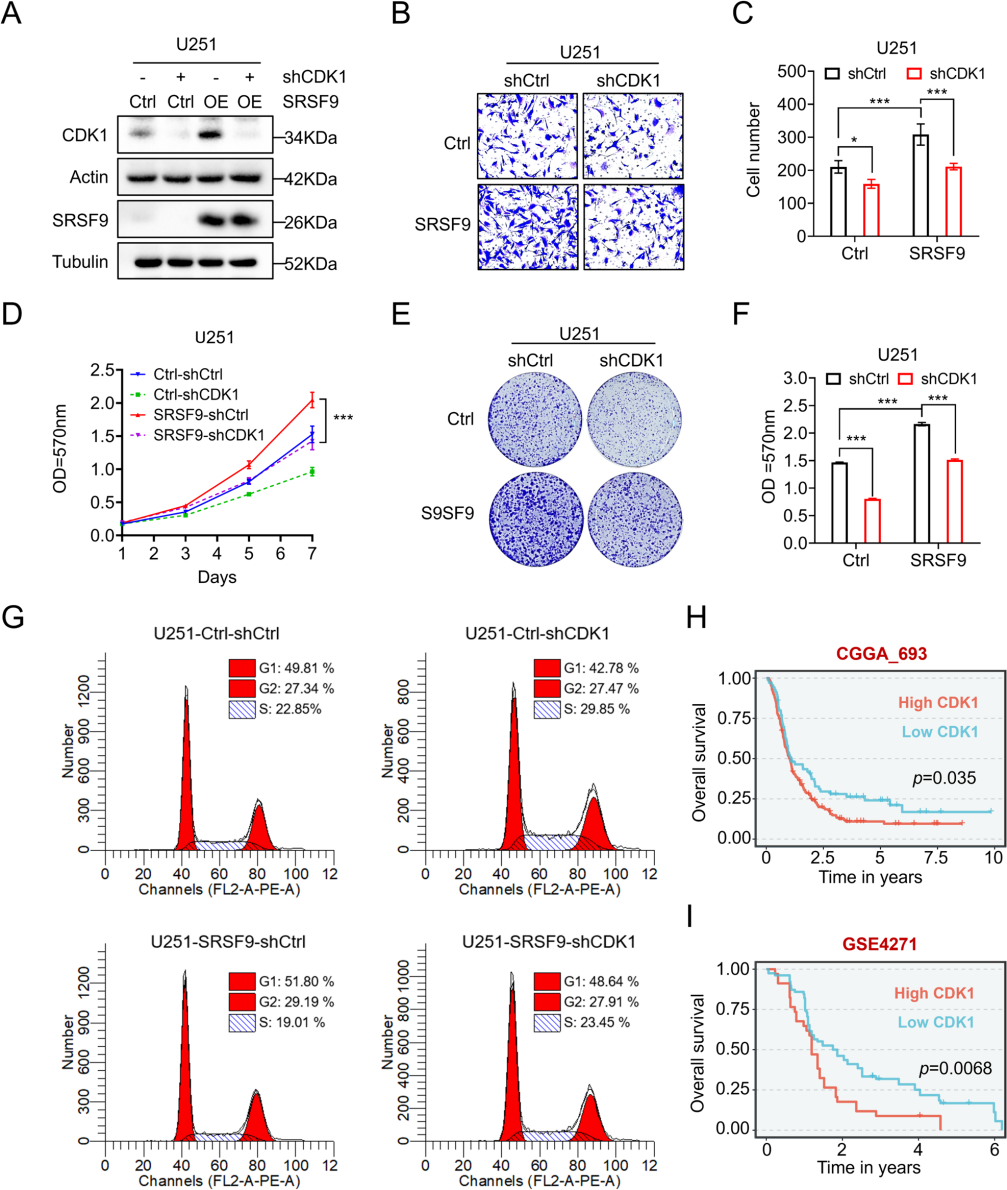
CDK1 participates in SRSF9-promoted cell proliferation and migration. **A** Western blotting assessed CDK1 protein levels in U251 cells with SRSF9 overexpression and CDK1 shRNA interference. **B**, **C** Transwell assay evaluated the effects of CDK1 shRNA on migration in SRSF9-overexpressing U251 cells. MTT assay **D** and colony formation assay **E**, **F** measured the effects of CDK1 shRNA on proliferation in U251 cells induced by SRSF9 overexpression. **G** Cell cycle analysis illustrated the impact of CDK1 shRNA on SRSF9-overexpressing U251 cells, detailing the proportion at each cell cycle stage. **H, I** Survival analysis, correlating CDK1 expression with overall survival in GBM patients, was based on CGGA (CGGA_693, *n* = 237), GEO (GSE4271, *n* = 152)

Given the facts that SRSF9 promote proliferation and migration of GBM cells (Fig. 3) and CDK1 expression is regulated by SRSF9 (Fig. 5), we wondered if CDK1 mediates the cellular functions of SRSF9. To this end, a shRNA targeting CDK1 was designed to construct the stable cell lines and the knockdown of CDK1 expression was confirmed by Western blot analyses (Fig. 6A). Then we evaluated the migration and proliferation capabilities of these cells. The results showed that shRNA-mediated CDK1 interference significantly diminished the enhanced proliferation and migration in SRSF9-overexpressing cells (Fig. 6B–F). Additionally, flow cytometry analyses demonstrated that SRSF9 overexpression accelerated the transition from S-phase to G2 in U251 cells, but this process was inhibited when CDK1 was knocked down, resulting in S-phase arrest (Fig. 6G). Furthermore, bioinformatics analyses of different public datasets revealed that GBM patients with increased CDK1 expression had reduced survival time (Fig. 6H, I), confirming the importance of CDK1 in regulating GBM progression. Together, SRSF9 can enhance CDK1 expression to promote cell proliferation and migration, thereby contributing to GBM development.

### SRSF9 regulates CDK1 transcription through binding to the promoter

**Fig. 7:**
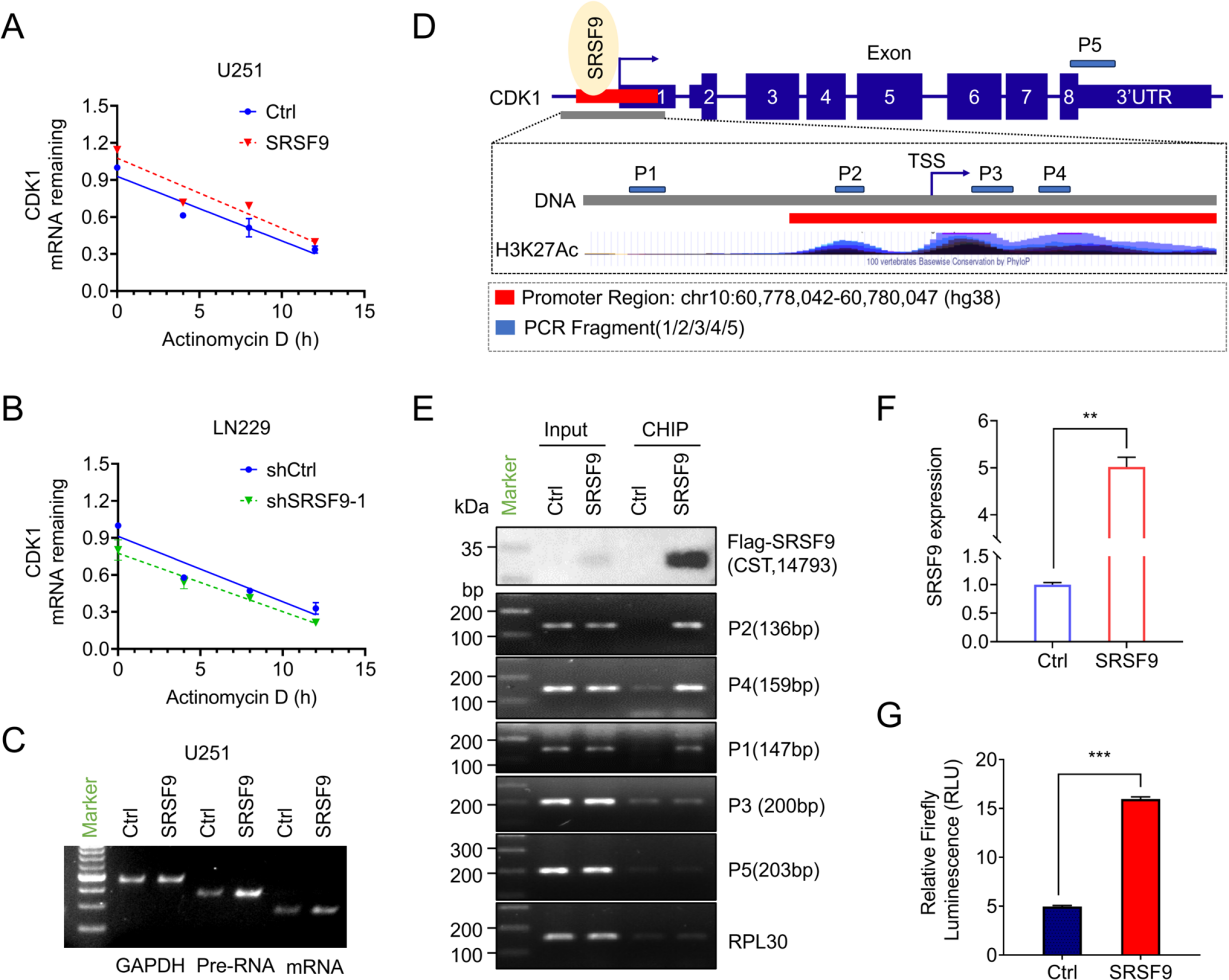
SRSF9 regulates CDK1 transcription through binding to the promoter. **A**, **B** Analysis of CDK1 mRNA stability: RT-qPCR was employed to assess the half-life of CDK1 mRNA (normalized to 0 h) in the context of SRSF9 overexpression and knockdown, following treatment with actinomycin D. **C** Semi-quantitative RT-PCR analysis measured the transcription rate of CDK1 mRNA and its precursor RNA following SRSF9 overexpression. **D** Schematic representation includes the CDK1 promoter region from −457 to + 1570 relative to the TSS, highlighting PCR fragments P1-P5: P1 outside the promoter, P2-P4 inside the promoter, and P5 near the 3' UTR. Primers for these fragments were designed to explore interactions with SRSF9. **E** The FLAG antibody detected FLAG-SRSF9 enrichment efficiency in ChIP assay (top). ChIP-PCR on U251 cells infected with SRSF9-overexpressing or control (Ctrl) viruses analyzed SRSF9's binding to the CDK1 promoter (bottom). **F** Quality control confirmed SRSF9 overexpression in HEK293T cells transfected with a dual-luciferase reporter gene. **G** CDK1 promoter activity assay: the CDK1 promoter was cloned into a dual-luciferase reporter plasmid to assess its activity in HEK293T cells with overexpressed SRSF9. Renilla luciferase was used as an internal control

Given the observed influence of SRSF9 on CDK1 expression, we hypothesized that SRSF9 may exert its effect on CDK1 mRNA stability, splicing efficiency, or transcription levels. To test these hypotheses, we first measured the half-lives of CDK1 mRNA in GBM cell lines with upregulated or downregulated SRSF9 expression, and found that SRSF9 has no significant impact on CDK1 mRNA stability (Fig. 7A, B). Then we measured pre-mRNA levels of CDK1 by semi-quantitative RT-PCR analyses in cells with or without SRSF9 overexpression, and found an increased CDK1 pre-mRNA level with SRSF9 overexpression, suggesting SRSF9 may regulate CDK1 expression at the transcriptional level (Fig. 7C, Table S1). Using ChIPBase (Huang et al. 2023) and UCSC’s H3K27Ac data, we identified the CDK1 gene promoter region and designed specific primers (Fig. 7D, Table S1) to verify protein-DNA interactions. The ChIP-PCR results demonstrated that SRSF9 was significantly enriched at the CDK1 promoter regions P1, P2 and P4 (Fig. 7E). Subsequently, the CDK1 gene promoter was cloned to the pGL3-basic vector for dual-luciferase assay. The results showed that SRSF9 overexpression significantly increased the luciferase activity, suggesting the transcriptional regulation of CDK1 expression by SRSF9 (Fig. 7F, G). Together, SRSF9 may regulates CDK1 expression at transcriptional level.

## Discussion

The SRSF protein family is shown to regulate gene transcription, RNA splicing, RNA export, RNA stability and protein translation (Zhong et al. 2009; Wagner and Frye 2021; Jin 2022), and their upregulation is associated with cancer development (Stanley and Abdel-Wahab 2022; An et al. 2024). In this study, we found an increased SRSF9 expression among different tumors, notably in GBM (Fig. 1), and the elevated SRSF9 expression closely correlates to poor survival of GBM patients (Fig. 2). Therefore, SRSF9 is pivotal in glioma development and could be a prognostic marker to predict patients’ survival.

Through gain- and loss-of-function approach, we demonstrated that SRSF9 accelerates GBM cell growth and migration (Fig. 3). These findings are consistent with what has been observed in hepatocellular carcinoma (HCC), where inhibition of SRSF9 resulted in reduced proliferation and migration (Liu et al. 2022b). SRSF9 has also been found to function as an m6A-binding protein in colorectal cancer (CRC), enhancing the stability of specific mRNAs involved in CRC malignant progression (Wang et al. 2022b). Thus, SRSF9 plays oncogenic roles in a wide spectrum of cancers.

SRSF9 co-expressed gene enrichment analysis through GSEA indicates its involvement in cell cycle regulation (Fig. 4). Through a correlation analysis of GBM tumor samples, along with cellular assays of SRSF9 knockdown and overexpression, we revealed that SRSF9 positively regulates the expression of CDK1 (Fig. 5), a cell cycle controller. Moreover, flow cytometry analyses showed that regulation of SRSF9 and CDK1 affected the S phase transition in U251 cells, suggesting that they have a regulatory role in cell cycle progression (Fig. 6G). Notably, CDK1 depletion disrupts S-phase arrest and ATR/ATM target phosphorylation after DNA damage (Satyanarayana et al. 2008; Johnson et al. 2009), which indirectly supports our conclusion. Since numerous studies have documented aberrant expression of CDK1 in various tumors, linking it to poor patient prognosis (Piao et al. 2019; Li et al. 2020; Du et al. 2023; Han et al. 2023), analyzing the impact of SRSF9 on S phase transition via CDK1 in GBM cells will deepen our molecular insights.

SRSF9, an RNA-binding protein, is reported to participate in mRNA splicing (Ha et al. 2021), protein export from the nucleus (Raffetseder et al. 2003), protein translation (Fu et al. 2013a; Matsumoto et al. 2021), and A-to-I editing (Shanmugam et al. 2018). Our findings through ChIP-PCR and dual-luciferase assays indicate that SRSF9 specifically targets the CDK1 gene promoter to enhance transcription, resulting in elevated CDK1 protein levels (Fig. 7). This finding highlights a novel involvement of SRSF9 in transcriptional regulation at the DNA level and suggests that its regulation of CDK1 may be associated with chromatin remodeling. Although chromatin remodeling is known to broadly affect cancer (Weissman and Knudsen 2009; Kadoch and Crabtree 2015), the specific role of SRSF9 in this process has not been clarified. Like transcription factors (TFs), RNA-binding proteins (RBPs) often target genomic hotspots, particularly gene promoters, where their binding significantly influences transcriptional activity (Xiao et al. 2019). Study reported DDX43, an RNA-binding protein, as pivotal in germ cell development through its role in chromatin remodeling (Tan et al. 2023), underscoring the importance of open chromatin for transcription factor access and gene regulation. Given that altered chromatin dynamics lead to an accelerated cell cycle, which in turn increases cancer risk (Ferraro 2016), it becomes crucial to further investigate how SRSF9 affects CDK1 and other genes. This research should delve into changes in chromatin accessibility and recruitment of transcription factors, using advanced techniques such as mass spectrometry and Assay for Transposase Accessible-Chromatin with high-throughput sequencing (ATAC-seq) for validation.

In summary, this study identifies significant upregulation of SRSF9 in GBM and demonstrates that its high expression correlates with poor survival of GBM patients. Utilizing gain-of-function and loss-of-function approaches, it was shown that SRSF9 plays a role in promoting proliferation and migration of GBM cells. Mechanistic analysis showed that SRSF9 binds to the promoter region of the CDK1 gene, leading to an increase in transcription levels and ultimately promoting GBM cell proliferation. These findings shed light on the cellular functions of SRSF9 in GBM and highlight its potential as a therapeutic target for GBM.

## Supplementary Information

Below is the link to the electronic supplementary material.

Supplementary file1 (DOCX 16244 KB)

Supplementary file2 (XLSX 511 KB)

## Data Availability

The datasets generated during and/or analyzed during the current study are available from the corresponding author on reasonable request.

## References

[CR1] An W, Yang Q, Xi Y, Pan H, Huang H, Chen Q (2024) Identification of SRSF10 as a promising prognostic biomarker with functional significance among SRSFs for glioma. Life Sci 338:122392. 10.1016/j.lfs.2023.122392 38160788 10.1016/j.lfs.2023.122392

[CR2] Barta A, Kalyna M, Reddy AS (2010) Implementing a rational and consistent nomenclature for serine/arginine-rich protein splicing factors (SR proteins) in plants. Plant Cell 22:2926–2929. 10.1105/tpc.110.078352 20884799 10.1105/tpc.110.078352 PMC2965536

[CR3] Ding L, Cao J, Lin W, Chen H, Xiong X, Ao H (2020) The roles of cyclin-dependent kinases in cell-cycle progression and therapeutic strategies in human breast cancer. Int J Mol Sci 21:1960. 10.3390/ijms21061960 32183020 10.3390/ijms21061960 PMC7139603

[CR4] Du Q, Liu W, Mei T, Wang J, Qin T, Huang D (2023) Prognostic and immunological characteristics of CDK1 in lung adenocarcinoma: a systematic analysis. Front Oncol 13:1128443. 10.3389/fonc.2023.1128443 36950551 10.3389/fonc.2023.1128443 PMC10025485

[CR5] Ferraro A (2016) Altered primary chromatin structures and their implications in cancer development. Cell Oncol (dordr) 39:195–210. 10.1007/s13402-016-0276-6 27007278 10.1007/s13402-016-0276-6 PMC13001864

[CR6] Fu Y, Huang B, Shi Z, Han J, Wang Y, Huangfu J (2013a) SRSF1 and SRSF9 RNA binding proteins promote Wnt signalling-mediated tumorigenesis by enhancing beta-catenin biosynthesis. EMBO Mol Med 5:737–750. 10.1002/emmm.201202218 23592547 10.1002/emmm.201202218 PMC3662316

[CR7] Fu Y, Huang B, Shi Z, Han J, Wang Y, Huangfu J (2013b) SRSF1 and SRSF9 RNA binding proteins promote Wnt signalling-mediated tumorigenesis by enhancing β-catenin biosynthesis. EMBO Mol Med 5:737–750 23592547 10.1002/emmm.201202218 PMC3662316

[CR8] Ghafouri-Fard S, Khoshbakht T, Hussen BM, Dong P, Gassler N, Taheri M (2022) A review on the role of cyclin dependent kinases in cancers. Cancer Cell Int 22:325. 10.1186/s12935-022-02747-z 36266723 10.1186/s12935-022-02747-z PMC9583502

[CR9] Ha J, Jang H, Choi N, Oh J, Min C, Pradella D (2021) SRSF9 regulates cassette exon splicing of caspase-2 by interacting with its downstream exon. Cells 10:679. 10.3390/cells10030679 33808656 10.3390/cells10030679 PMC8003524

[CR10] Han Z, Jia Q, Zhang J, Chen M, Wang L, Tong K (2023) Deubiquitylase YOD1 regulates CDK1 stability and drives triple-negative breast cancer tumorigenesis. J Exp Clin Cancer Res 42:228. 10.1186/s13046-023-02781-3 37667382 10.1186/s13046-023-02781-3 PMC10478497

[CR11] Huang J, Zheng W, Zhang P, Lin Q, Chen Z, Xuan J (2023) ChIPBase v3.0: the encyclopedia of transcriptional regulations of non-coding RNAs and protein-coding genes. Nucleic Acids Res 51:D46–D56. 10.1093/nar/gkac1067 36399495 10.1093/nar/gkac1067 PMC9825553

[CR12] Janjua TI, Rewatkar P, Ahmed-Cox A, Saeed I, Mansfeld FM, Kulshreshtha R (2021) Frontiers in the treatment of glioblastoma: past, present and emerging. Adv Drug Deliv Rev 171:108–138. 10.1016/j.addr.2021.01.012 33486006 10.1016/j.addr.2021.01.012

[CR13] Jin X (2022) Regulatory network of serine/arginine-rich (SR) proteins: the molecular mechanism and physiological function in plants. Int J Mol Sci 23:10147. 10.3390/ijms231710147 36077545 10.3390/ijms231710147 PMC9456285

[CR14] Johnson N, Cai D, Kennedy RD, Pathania S, Arora M, Li YC (2009) Cdk1 participates in BRCA1-dependent S phase checkpoint control in response to DNA damage. Mol Cell 35:327–339. 10.1016/j.molcel.2009.06.036 19683496 10.1016/j.molcel.2009.06.036 PMC3024055

[CR15] Kadoch C, Crabtree GR (2015) Mammalian SWI/SNF chromatin remodeling complexes and cancer: mechanistic insights gained from human genomics. Sci Adv 1:e1500447. 10.1126/sciadv.1500447 26601204 10.1126/sciadv.1500447 PMC4640607

[CR16] King JL, Benhabbour SR (2021) Glioblastoma multiforme-a Look at the past and a glance at the future. Pharmaceutics 13:1053. 10.3390/pharmaceutics13071053 34371744 10.3390/pharmaceutics13071053 PMC8309001

[CR17] Li JH, Liu S, Zhou H, Qu LH, Yang JH (2014) starBase v2.0: decoding miRNA-ceRNA, miRNA-ncRNA and protein-RNA interaction networks from large-scale CLIP-Seq data. Nucleic Acids Res 42:D92-97. 10.1093/nar/gkt1248 24297251 10.1093/nar/gkt1248 PMC3964941

[CR18] Li J, Wang Y, Wang X, Yang Q (2020) CDK1 and CDC20 overexpression in patients with colorectal cancer are associated with poor prognosis: evidence from integrated bioinformatics analysis. World J Surg Oncol 18:50. 10.1186/s12957-020-01817-8 32127012 10.1186/s12957-020-01817-8 PMC7055103

[CR19] Li Y, Dou Y, Da Veiga LF, Geffen Y, Calinawan AP, Aguet F (2023) Proteogenomic data and resources for pan-cancer analysis. Cancer Cell 41:1397–1406. 10.1016/j.ccell.2023.06.009 37582339 10.1016/j.ccell.2023.06.009 PMC10506762

[CR20] Liberzon A, Subramanian A, Pinchback R, Thorvaldsdottir H, Tamayo P, Mesirov JP (2011) Molecular signatures database (MSigDB) 3.0. Bioinformatics 27:1739–1740. 10.1093/bioinformatics/btr260 21546393 10.1093/bioinformatics/btr260 PMC3106198

[CR21] Liberzon A, Birger C, Thorvaldsdottir H, Ghandi M, Mesirov JP, Tamayo P (2015) The molecular signatures database (MSigDB) hallmark gene set collection. Cell Syst 1:417–425. 10.1016/j.cels.2015.12.004 26771021 10.1016/j.cels.2015.12.004 PMC4707969

[CR22] Liu J, Wang Y, Yin J, Yang Y, Geng R, Zhong Z (2022a) Pan-cancer analysis revealed SRSF9 as a new biomarker for prognosis and immunotherapy. J Oncol 2022:3477148. 10.1155/2022/3477148 35069733 10.1155/2022/3477148 PMC8769850

[CR23] Liu Y, Mao X, Ma Z, Chen W, Guo X, Yu L (2022b) Aberrant regulation of LncRNA TUG1-microRNA-328-3p-SRSF9 mRNA Axis in hepatocellular carcinoma: a promising target for prognosis and therapy. Mol Cancer 21:36. 10.1186/s12943-021-01493-6 35120500 10.1186/s12943-021-01493-6 PMC8815183

[CR24] Liu A, Jiang B, Song C, Zhong Q, Mo Y (2023a) Isoliquiritigenin inhibits circ0030018 to suppress glioma tumorigenesis via the miR-1236/HER2 signaling pathway. MedComm 4:e282. 10.1002/mco2.282 37250146 10.1002/mco2.282 PMC10220153

[CR25] Liu Z, Liu L, Weng S, Xu H, Xing Z, Ren Y (2023b) BEST: a web application for comprehensive biomarker exploration on large-scale data in solid tumors. J Big Data. 10.1186/s40537-023-00844-y37193361

[CR26] Matsumoto E, Matsumoto Y, Inoue J, Yamamoto Y, Suzuki T (2021) AMP-activated protein kinase regulates beta-catenin protein synthesis by phosphorylating serine/arginine-rich splicing factor 9. Biochem Biophys Res Commun 534:347–352. 10.1016/j.bbrc.2020.11.079 33248688 10.1016/j.bbrc.2020.11.079

[CR27] Piao J, Zhu L, Sun J, Li N, Dong B, Yang Y (2019) High expression of CDK1 and BUB1 predicts poor prognosis of pancreatic ductal adenocarcinoma. Gene 701:15–22. 10.1016/j.gene.2019.02.081 30898709 10.1016/j.gene.2019.02.081

[CR28] Raffetseder U, Frye B, Rauen T, Jurchott K, Royer HD, Jansen PL (2003) Splicing factor SRp30c interaction with Y-box protein-1 confers nuclear YB-1 shuttling and alternative splice site selection. J Biol Chem 278:18241–18248. 10.1074/jbc.M212518200 12604611 10.1074/jbc.M212518200

[CR29] Satyanarayana A, Hilton MB, Kaldis P (2008) p21 Inhibits Cdk1 in the absence of Cdk2 to maintain the G1/S phase DNA damage checkpoint. Mol Biol Cell 19:65–77. 10.1091/mbc.e07-06-0525 17942597 10.1091/mbc.E07-06-0525 PMC2174178

[CR30] Schaff LR, Mellinghoff IK (2023) Glioblastoma and other primary brain malignancies in adults: a review. JAMA 329:574–587. 10.1001/jama.2023.0023 36809318 10.1001/jama.2023.0023 PMC11445779

[CR31] Shanmugam R, Zhang F, Srinivasan H, Charles Richard JL, Liu KI, Zhang X (2018) SRSF9 selectively represses ADAR2-mediated editing of brain-specific sites in primates. Nucleic Acids Res 46:7379–7395. 10.1093/nar/gky615 29992293 10.1093/nar/gky615 PMC6101530

[CR32] Stanley RF, Abdel-Wahab O (2022) Dysregulation and therapeutic targeting of RNA splicing in cancer. Nat Cancer 3:536–546. 10.1038/s43018-022-00384-z 35624337 10.1038/s43018-022-00384-z PMC9551392

[CR33] Subramanian A, Tamayo P, Mootha VK, Mukherjee S, Ebert BL, Gillette MA (2005) Gene set enrichment analysis: a knowledge-based approach for interpreting genome-wide expression profiles. Proc Natl Acad Sci U S A 102:15545–15550. 10.1073/pnas.0506580102 16199517 10.1073/pnas.0506580102 PMC1239896

[CR34] Tan AC, Ashley DM, Lopez GY, Malinzak M, Friedman HS, Khasraw M (2020) Management of glioblastoma: state of the art and future directions. CA Cancer J Clin 70:299–312. 10.3322/caac.21613 32478924 10.3322/caac.21613

[CR35] Tan H, Wang W, Zhou C, Wang Y, Zhang S, Yang P (2023) Single-cell RNA-seq uncovers dynamic processes orchestrated by RNA-binding protein DDX43 in chromatin remodeling during spermiogenesis. Nat Commun 14:2499. 10.1038/s41467-023-38199-w 37120627 10.1038/s41467-023-38199-w PMC10294715

[CR36] Tang Z, Kang B, Li C, Chen T, Zhang Z (2019) GEPIA2: an enhanced web server for large-scale expression profiling and interactive analysis. Nucleic Acids Res 47:W556–W560. 10.1093/nar/gkz430 31114875 10.1093/nar/gkz430 PMC6602440

[CR37] Wagner RE, Frye M (2021) Noncanonical functions of the serine-arginine-rich splicing factor (SR) family of proteins in development and disease. BioEssays 43:e2000242. 10.1002/bies.202000242 33554347 10.1002/bies.202000242

[CR38] Wang R, Su Q, Yin H, Wu D, Lv C, Yan Z (2021) Inhibition of SRSF9 enhances the sensitivity of colorectal cancer to erastin-induced ferroptosis by reducing glutathione peroxidase 4 expression. Int J Biochem Cell Biol 134:105948. 10.1016/j.biocel.2021.105948 33609745 10.1016/j.biocel.2021.105948

[CR39] Wang C, Liu WR, Tan S, Zhou JK, Xu X, Ming Y (2022a) Characterization of distinct circular RNA signatures in solid tumors. Mol Cancer 21:63. 10.1186/s12943-022-01546-4 35236349 10.1186/s12943-022-01546-4 PMC8889743

[CR40] Wang X, Lu X, Wang P, Chen Q, Xiong L, Tang M (2022b) SRSF9 promotes colorectal cancer progression via stabilizing DSN1 mRNA in an m6A-related manner. J Transl Med 20:198. 10.1186/s12967-022-03399-3 35509101 10.1186/s12967-022-03399-3 PMC9066907

[CR41] Weissman B, Knudsen KE (2009) Hijacking the chromatin remodeling machinery: impact of SWI/SNF perturbations in cancer. Cancer Res 69:8223–8230. 10.1158/0008-5472.CAN-09-2166 19843852 10.1158/0008-5472.CAN-09-2166 PMC3272668

[CR42] Xiao R, Chen JY, Liang Z, Luo D, Chen G, Lu ZJ (2019) Pervasive chromatin-RNA binding protein interactions enable RNA-based regulation of transcription. Cell 178(107–121):e118. 10.1016/j.cell.2019.06.001 10.1016/j.cell.2019.06.001 PMC6760001 31251911

[CR43] Zhao Z, Zhang KN, Wang Q, Li G, Zeng F, Zhang Y (2021) Chinese Glioma Genome Atlas (CGGA): A comprehensive resource with functional genomic data from Chinese glioma patients. Genomics Proteomics Bioinformatics 19:1–12. 10.1016/j.gpb.2020.10.005 33662628 10.1016/j.gpb.2020.10.005 PMC8498921

[CR44] Zhong XY, Wang P, Han J, Rosenfeld MG, Fu XD (2009) SR proteins in vertical integration of gene expression from transcription to RNA processing to translation. Mol Cell 35:1–10. 10.1016/j.molcel.2009.06.016 19595711 10.1016/j.molcel.2009.06.016 PMC2744344

