# Supplementary material for: SRSF9 promotes cell proliferation and migration of glioblastoma through enhancing CDK1 expression
Source: J Cancer Res Clin Oncol. 2024 Jun 6;150(6):292. doi: 10.1007/s00432-024-05797-0 (PMC11156731; doi:10.1007/s00432-024-05797-0)
Supplement: Supplementary file 1 — Supplementary file1 (DOCX 16244 KB) [file 432_2024_5797_MOESM1_ESM.docx]

SRSF9 promotes cell proliferation and migration of glioblastoma through enhancing CDK1 expression

Chunyuan Luo ^1,^ ^†^, Juan He ^1,^ ^†,^ *, Yang Yang ^1^, Ke Wu ^1^, Xin Fu ^1^, Jian Cheng ^2^, Yue Ming ^1^, Wenrong Liu ^1^, Yong Peng ^1,^ *

^1^ Laboratory of Molecular Oncology, Frontiers Science Center for Disease-related Molecular Network, State Key Laboratory of Biotherapy, West China Hospital, Sichuan University, Chengdu 610041, China.

^2^ Department of Neurosurgery, West China Hospital, Sichuan University, Chengdu, 610041, China.

* Corresponding author: Juan He (hejuan@scu.edu.cn); Yong Peng (yongpeng@scu.edu.cn).

† Chunyuan Luo and Juan He have contributed equally to this work.

**Supplementary figures**


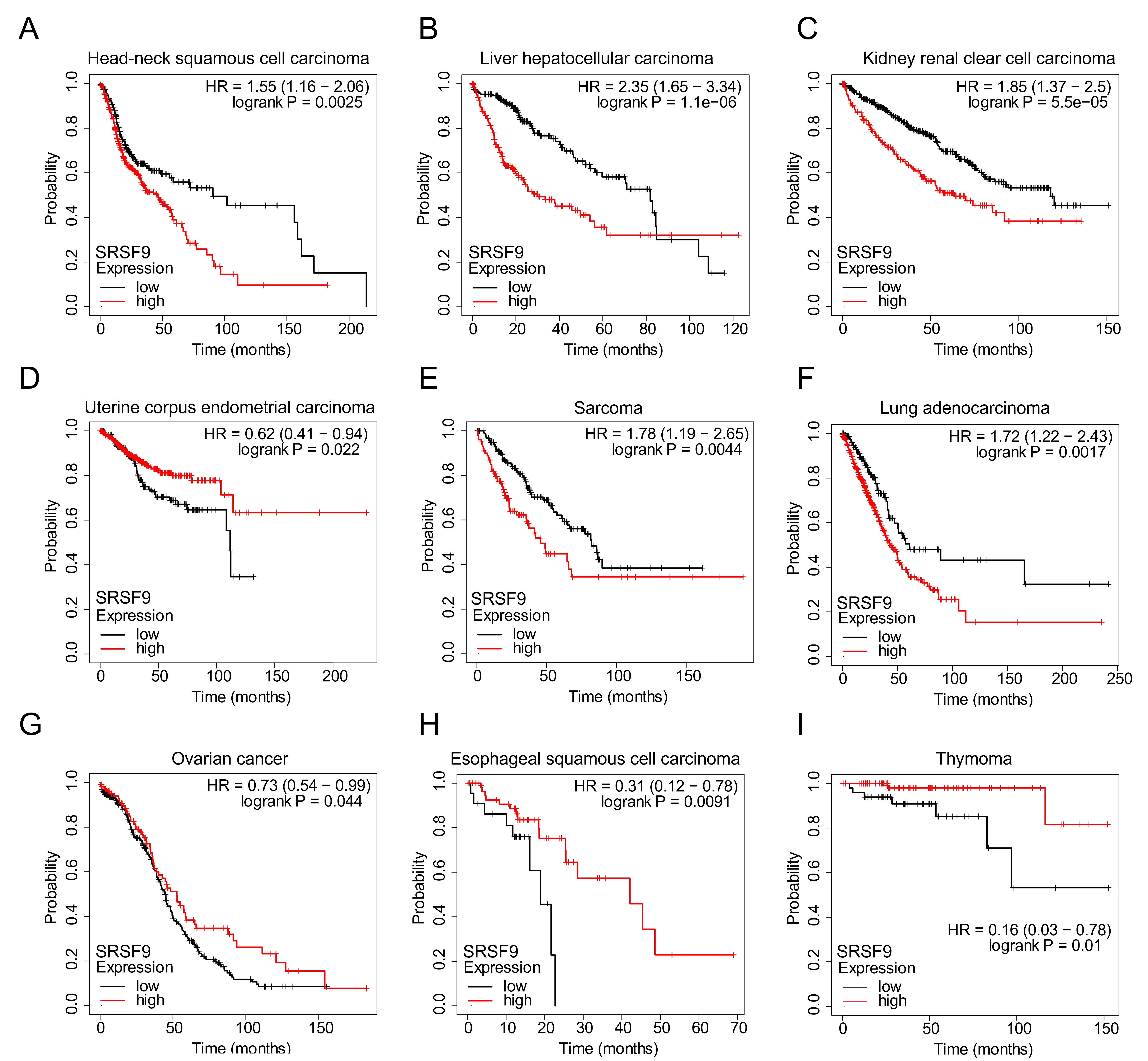


**Figure S1** Prognostic impact of SRSF9 in various cancers assessed through survival analysis using the Kaplan–Meier plotter database, covering: (**A**) Head-neck squamous cell carcinoma (n=499), (**B**) Liver hepatocellular carcinoma (n=370), (**C**) Kidney renal clear cell carcinoma (n=530), (**D**) Uterine corpus endometrial carcinoma (n=542), (**E**) Sarcoma (n=259), (**F**) Lung adenocarcinoma (n=504), (**G**) Ovarian cancer (n=373), (**H**) Esophageal squamous cell carcinoma (n=81) and (**I)** Thymoma (n=118).


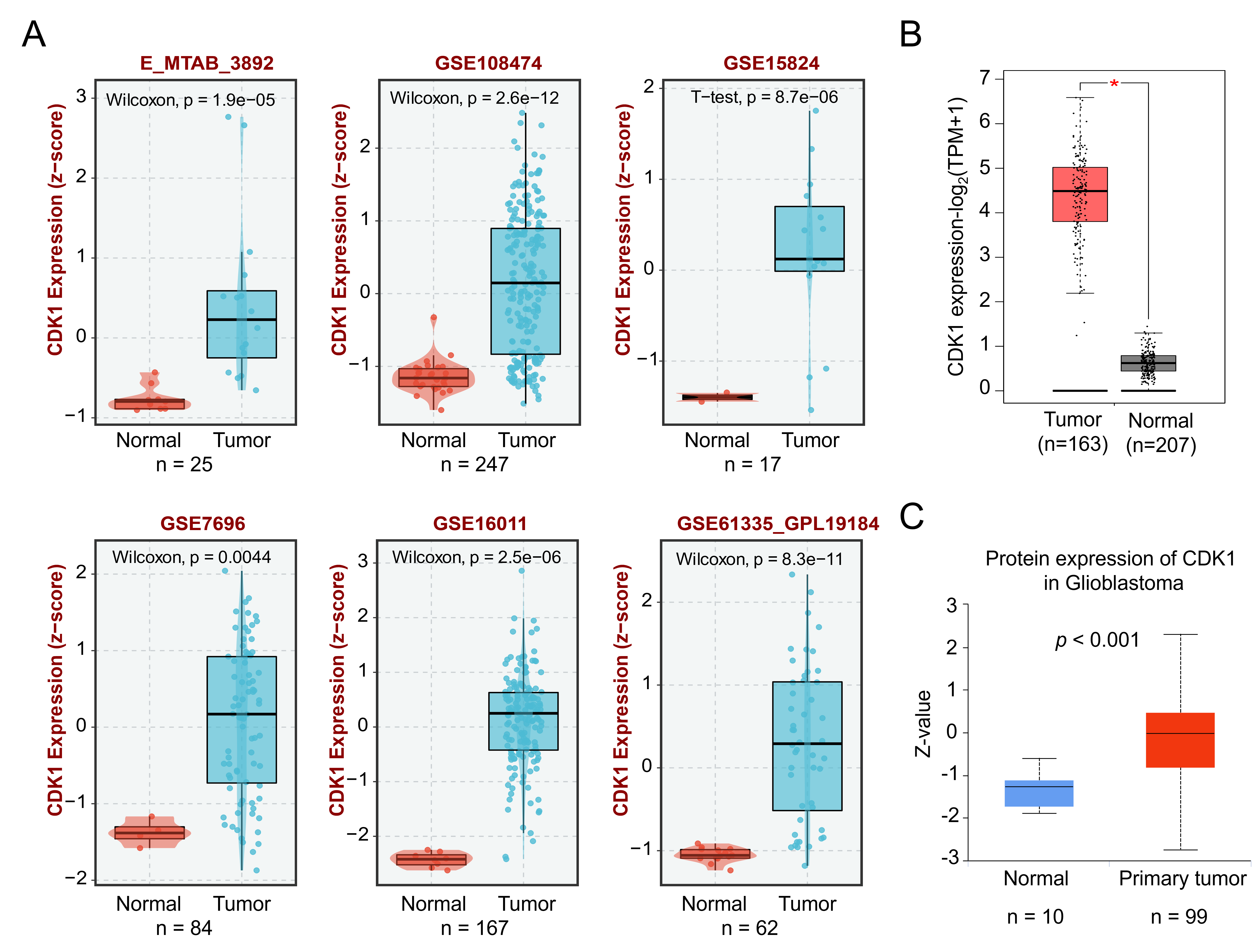


**Figure S2** CDK1 is upregulated in GBM. (**A**) CDK1 expression analyzed in six independent GEO cohorts. Including: E_MTAB_3892 (n=25), GSE108474 (n=247), GSE15824 (n=17), GSE7696 (n=84), GSE16011 (n=167), GSE61335_GPL19184 (n=62). (**B**) CDK1 mRNA expression in GBM patients was analyzed using the GEPIA2 database. (**C**) A box plot compares CDK1 protein expression in GBM (99 samples) versus normal tissues (10 samples) using UALCAN data.


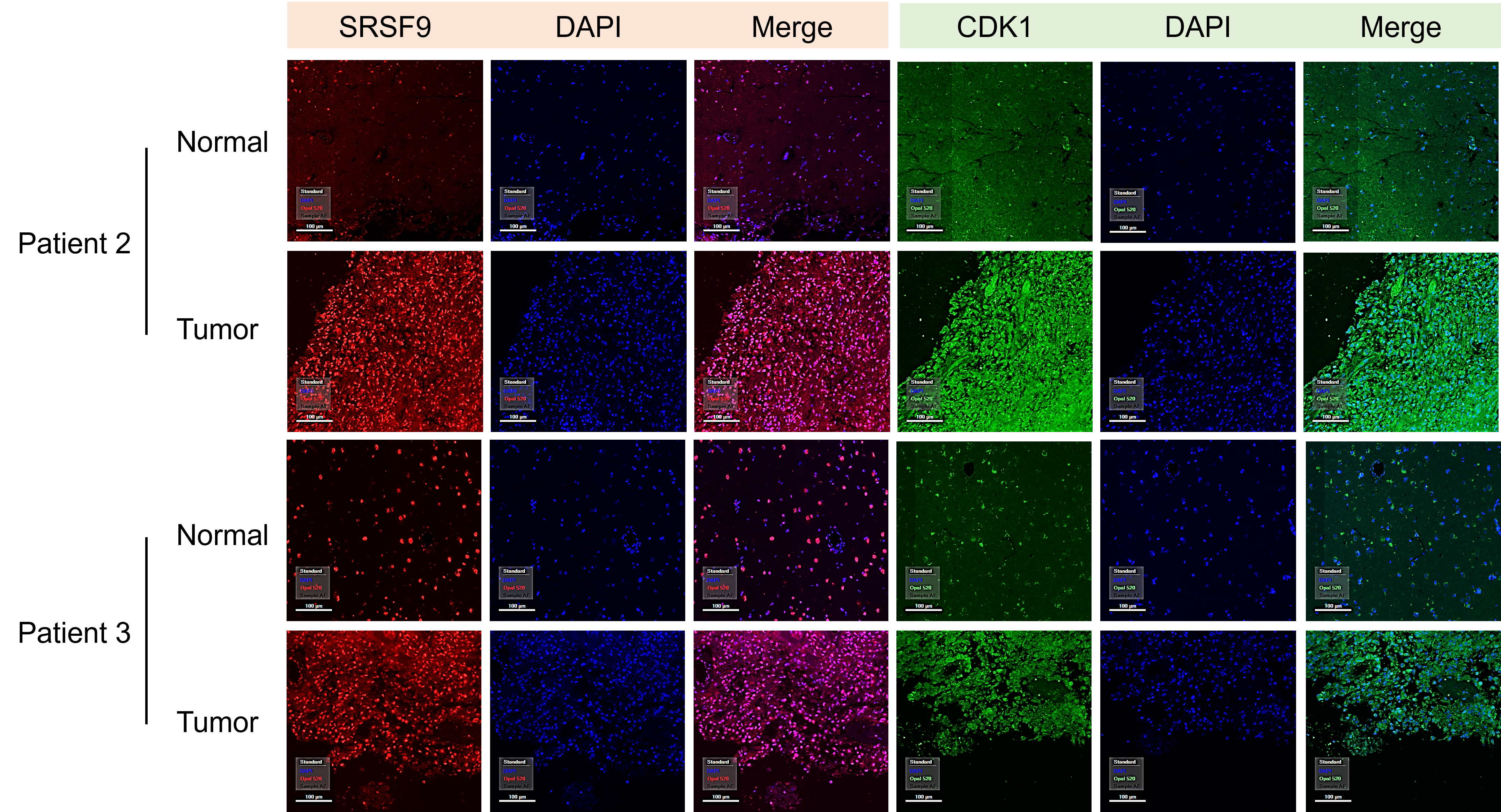


**Figure S3** Immunofluorescence assay quantified the levels of SRSF9 and CDK1 in GBM tissues (Tumor) and adjacent normal tissues (Normal) from two patients.
